# Supplementary material for: Phenolic Compounds from Mori Cortex Ameliorate Sodium Oleate-Induced Epithelial–Mesenchymal Transition and Fibrosis in NRK-52e Cells through CD36
Source: Molecules. 2021 Oct 11;26(20):6133. doi: 10.3390/molecules26206133 (PMC8540367; doi:10.3390/molecules26206133)
Supplement: Supplementary file 1 [file molecules-26-06133-s001.zip › molecules-1384799-supplementary.pdf]

1. The effect of silencing CD36 on NRK-52e cells

The results showed that the level of CD36 mRNA in the siCD36 group was extremely significantly lower than NC group (P < 0.01), as shown in Figure S1A. We detected the expression of CD36 protein and found that compared with NC group, the relative expression of CD36 in M group was significantly increased. Compared with M group, the relative expression of CD36 protein in Y-1 and Y-2 groups was significantly down-regulated. Compared with the NC-siCD36 group, the relative expression of CD36 protein in the M-siCD36 group was significantly reduced. Compared with the M-siCD36 group, the Y-1-siCD36 and Y-2-siCD36 groups had no significant changes. This showed that siCD36 can make the amelioration effect of Y-1 and Y-2 disappear.

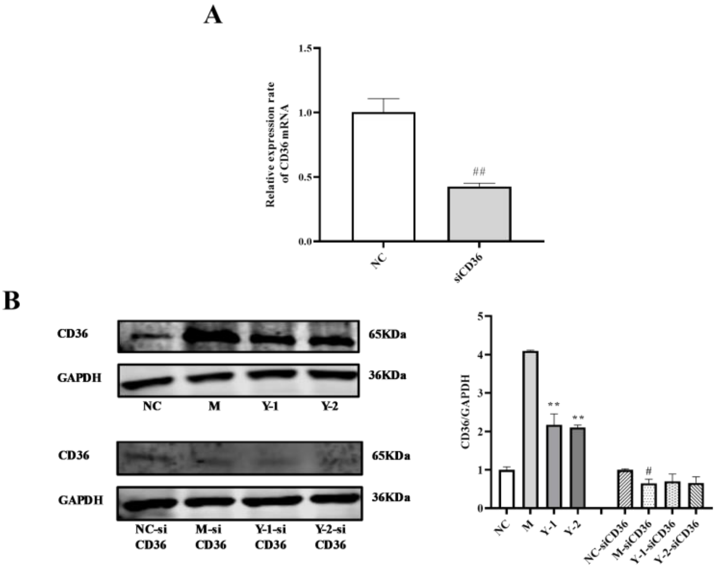

Figure S1. Effect of CD36 silencing on NRK-52e cells. The expression of CD36 mRNA in NRK-52e cells were quantified by qRT-PCR after CD36 silencing(n = 3) (A); The expression of CD36 protein in NRK-52e cells were quantified by western bolt and normalized (n = 3) (B). ##P < 0.01 compared with NC group, **P<0.01 compared with M group.

2. The effect of cell viability on NRK-52e cells induced by Sodium Oleate

The results showed that sodium oleate has no inhibitory effect on NRK-52e compared with the NC group. Y-1 and Y-2 have a trend of improvement compared with the M group. It is suggested that sodium oleate has a certain inhibitory effect on NRK-52e, and the damage is not significant.

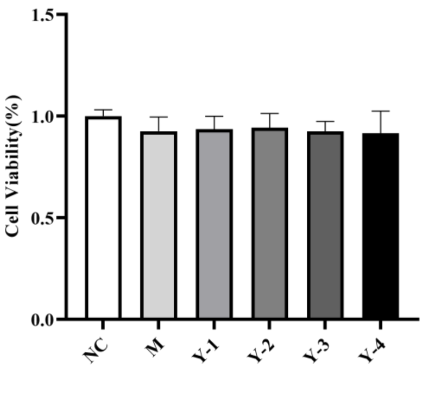

Figure S2. Effect of cell viability on NRK-52e cells induced by Sodium Oleate.
